# The role of *AIP* variants in pituitary adenomas and concomitant thyroid carcinomas in the Netherlands: a nationwide pathology registry (PALGA) study

**DOI:** 10.1007/s12020-020-02303-7

**Published:** 2020-04-24

**Authors:** E. C. Coopmans, A. Muhammad, A. F. Daly, W. W. de Herder, F. J. van Kemenade, A. Beckers, M. de Haan, A. J. van der Lely, E. Korpershoek, S. J. C. M. M. Neggers

**Affiliations:** 1 Department of Medicine, Endocrinology section, Pituitary Center Rotterdam, Erasmus University Medical Center, Rotterdam, The Netherlands; 2 Department of Endocrinology, Centre Hospitalier Universitaire de Liege, University of Liege, 4000 Liege, Belgium; 3 Department of Pathology, Erasmus Medical Center Cancer Institute, Erasmus University Medical Center, Rotterdam, The Netherlands

**Keywords:** Pituitary tumors, Thyroid carcinoma, AIP, Acromegaly

## Abstract

**Purpose:**

Germline mutations in the *aryl-hydrocarbon receptor interacting protein* (*AIP*) have been identified often in the setting of familial isolated pituitary adenoma (FIPA). To date there is no strong evidence linking germline *AIP* mutations to other neoplasms apart from the pituitary. Our primary objective was to investigate the prevalence of *AIP* gene mutations and mutations in genes that have been associated with neuroendocrine tumors in series of tumors from patients presenting with both pituitary adenomas and differentiated thyroid carcinomas (DTCs).

**Methods:**

Pathology samples were retrieved from all pituitary adenomas in patients with concomitant DTCs, including one with a known germline *AIP* variant. Subsequently, two additional patients with known germline *AIP* variants were included, of which one presented only with a follicular thyroid carcinoma (FTC).

**Results:**

In total, 17 patients (14 DTCs and 15 pituitary adenomas) were investigated by targeted next generation sequencing (NGS). The pituitary tumor samples revealed no mutations, while among the thyroid tumor samples *BRAF* (6/14, 42.9%) was the most frequently mutated gene, followed by *NRAS* (3/11, 27.3%). In one *AIP*-mutated FIPA kindred, the *AIP*-variant c.853C>T; p.Q285* was confirmed in the FTC specimen, including evidence of loss of heterozygosity (LOH) at the *AIP* locus in the tumor DNA.

**Conclusion:**

Although most observed variants in pituitary adenomas and DTCs were similar to those of sporadic DTCs, we confirmed in one *AIP* mutation-positive case the *AIP*-variant and LOH at this locus in an FTC specimen, which raises the potential role of the *AIP* mutation as a rare initiating event.

## Introduction

Pituitary adenomas are mostly benign monoclonal neoplasms that arise from any of the five hormone-secreting cell types of the anterior lobe of the pituitary gland, and cause disease due to hormonal hypersecretion and tumor mass effects. Most pituitary adenomas occur sporadically (95%). Although in the majority of these sporadic cases the exact molecular pathogenesis remains unknown, in a significant proportion of somatotropinomas (30%) and corticotropinomas (60%) activating somatic mutations have been found in the *GNAS* and *USP8* genes, respectively [1, 2]. In addition, germline mutations may predispose to pituitary tumorigenesis, which together represent about 5% of patients with pituitary adenomas [3].

Germline mutations have been described in the *aryl-hydrocarbon receptor interacting protein* (*AIP*) gene in the setting of either familial isolated pituitary adenoma (FIPA) or in simplex, young-onset pituitary adenomas, such as pituitary gigantism [4–6]. The *AIP* gene encodes a 330-amino-acid co-chaperone involved in subcellular trafficking, nuclear receptor stability, and transactivation potential [4, 7]. It is postulated that in *AIP*-mutated pituitary adenomas, *AIP* loses its activity as a tumor suppressor, which is supported by the association of loss-of-function mutations and the presence of loss of heterozygosity (LOH) at the *AIP* locus in the pituitary adenoma. To date there is no strong evidence linking germline *AIP* mutations to other neuroendocrine neoplasms apart from the pituitary.

The frequency of differentiated thyroid carcinomas (DTCs) is increased in patients with somatotropinomas, with papillary thyroid carcinoma (PTC) being the most frequently reported type (up to 25%) [8–13]. As thyroid follicular epithelial cells express insulin-like growth factor 1 (IGF-1) receptors and IGF-1 is an important factor for promoting replication and reducing apoptosis of these cells [14], IGF-1 could potentially be linked to the promotion of thyroid cancer in acromegalic patients. *BRAF* mutations have proved to be the most common genetic event (about 60% of cases) involved in the onset of PTC in the general population [15]; other frequently identified genetic events include point mutations of the *RAS* genes and *RET/PTC* and *PAX8/PPARɣ* chromosomal rearrangements [15, 16]. Based on earlier reports, LOH of chromosome 22 is particularly common in follicular thyroid carcinomas (FTCs), and it is associated with the widely invasive type [17–19].

Given the frequency of malignant thyroid tumors in somatotropinomas, the potential for a common mechanism behind both tumors remains valid. The role of an *AIP* mutation as an initiating event is open to question since the *AIP* protein may interact with the tyrosine kinase receptor, encoded by the *RET* protooncogene in the pituitary [20–24]. Coexistence of PTCs with somatotropinomas in *AIP*-mutated patients is very rare and has been described in three cases [25, 26]. Although only one case of LOH at the *AIP* locus (11q13) in FTCs is previously described [27], Daly et al. recently described an FTC in a teenaged *AIP* mutation-positive carrier in which decreased *AIP* staining was seen in the FTC tumor that was accompanied by LOH at the *AIP* locus in the tumor DNA [28]. Thus, the finding of DTCs and pituitary adenomas in the same individuals or kindreds could represent a rare association of germline *AIP* mutations.

To date, there has only been one study that reported in 12 patients with somatotropinomas and concomitant DTC that *AIP* was not overexpressed in the thyroid tumor tissue using immunohistochemistry [29]. Here we studied the presence of mutations in *AIP* in patients with DTCs and concomitant pituitary adenomas, including all five adenoma types. Subsequently, the available tumors from these patients were investigated using targeted next generation sequencing (NGS) for mutations in *AIP* and additional neuroendocrine tumor-related genes. Since these features are relative rare, a nationwide survey was performed in the Netherlands.

## Materials and methods

### Patients

From *Pathologisch-Anatomisch Landelijk Geautomatiseerd Archief* (PALGA), the nationwide Dutch network and registry of histo- and cytopathology, all patient records of individuals included 1993–2016 were retrieved matching the following search criteria: pituitary adenoma (i.e., prolactinomas, nonfunctioning pituitary adenomas (NFPAs), somatotropinomas, corticotropinomas, and thyrotropinomas) and DTC (i.e., FTC, follicular variant of papillary thyroid carcinoma (FVPTC), and PTC). The standardized records contain an encrypted patient identification number (allowing for identification of multiple samples of one patient), data on age at diagnosis and sex, date of arrival of the histological tissue, presence of metastasis, and the diagnosis of the pathology report.

The PALGA search identified 15 patients with a history of thyroid carcinoma and pituitary adenoma with no known genetic background (i.e., sporadic), except for one with a known germline *AIP* variant from the Erasmus University Medical Center that was part of the PALGA search data range as well. Two additional patients from this center with known germline *AIP* variants were included in the study, of which one who presented only with an FTC. The latter has a familial history of pituitary adenomas (i.e., father was *AIP* mutation carrier and diagnosed with acromegaly), however, the pituitary gland was not affected in this patient. Therefore, this patient was not identified in the PALGA search data range. The second patient with a somatotropinoma and classical-variant PTC was successfully treated by total thyroidectomy in 1975, and therefore not part of the PALGA search date range, while the tumor sample showed well-preserved histomorphology. In total, we included 17 patients.

Approval from the Medical Ethical Committee of the Erasmus University Medical Center and informed consent to use the tumor tissues for research purposes were obtained. Tumor tissues from all Dutch medical centers were used according to the code of conduct, Proper Secondary Use of Human Tissue, established by the Dutch Federation of Medical Scientific Societies [30].

### Data collection

Anonymized data were collected on age at diagnosis, sex, year of diagnosis, presence of metastasis, immunohistochemical staining results (adrenocorticotropic hormone (ACTH), follicle-stimulating hormone (FSH), GH, luteinizing hormone (LH), PRL) and type of DTC (FTC, FVPTC, or classical-variant PTC).

### Genetic analysis of germline AIP mutation

As mentioned above, three patients from the Erasmus University Medical Center included in the study were previously investigated for the presence of *AIP* mutations. This was performed using leukocyte DNA extracted from peripheral blood as described by Vierimaa et al. [5]; multiplex ligation-dependent probe amplification studies were performed as described previously [31, 32]. Normal population genetic databases were assessed for the presence of *AIP* variant frequencies; AIP variant pathogenicity was assessed using Alamut (Interactive Biosoftware). In addition, classification of variants was also performed according reported guidelines [33]. All patients provided informed written consent for genetic testing.

### Tumor DNA samples

We excluded low quality tissue of pituitary adenoma (*n* = 2) or thyroid carcinoma (*n* = 2) from the study. As mentioned before, patient no. 17 presented only with an FTC. In total 29 tumor DNA samples from 17 index patients were studied; DNA obtained exclusively from thyroid tumor was available for 14 (82.4%) of the cases, and only pituitary tumor DNA for 15 (88.2%) of the cases.

DNA was isolated from representative tumor areas by microdissection, from ~10 hematoxylin stained sections from formalin-fixed, paraffin-embedded (FFPE) tumor tissue, using proteinase-K and 5% Chelex 100 resin. Selection of representative tumor areas was performed on a paraffin slide stained with hematoxylin and eosin by a pathologist (L.O. and F.G.). In addition, DNA was quantified with the Quant-iT PicoGreen dsDNA Assay Kit (Thermo Fisher Scientific, Waltham, MA). All tumor DNAs that were used for mutation screening contained ≥60% of tumor cells.

### Targeted NGS and data analysis

A custom-made targeted gene panel (TGP) was designed using the TruSeq Custom Amplicon 1.5 kit system (Illumina, San Diego, CA) and the Ion AmpliSeq designer software (https://ampliseq.com/; Thermo Fisher Scientific, Breda, the Netherlands), to study DNA from FFPE tumor tissues (Table 1). The panel was designed specifically for FFPE-DNA use (amplicon range 125–175 bp). Targeting contained the entire coding sequences of *AIP* (coverage based on design: 92.11%), *CDKN1B* (96.84%)*, GNAS* (82.93%), *GPR101* (97.46%)*, HRAS* (63.08%)*, KRAS* (82.28%), *MEN1* (84.73%)*, NRAS* (100.00%)*, PIK3CA* (96.55%)*, PRKACB* (91.46%), *PRKAR1A* (100.00%), *RET* (86.34%)*, SDHA* (93.48%)*, SDHAF2* (100.00%), *SDHB* (98.65%), *SDHC* (91.93%)*, SDHD* (77.95%), and the hotspot region *BRAF (*p.V600E). In addition, single-nucleotide polymorphisms (SNPs) were selected on chromosome 11 and 22 to enable copy number variation (CNV) detection (Table 1). Mutation detection was performed using the S5-XL system (Ion Torrent) with manufacturer’s materials and protocols (Thermo Fisher Scientific). Library preparations and sequencing was performed as described earlier [34]. Data analysis was performed using SeqPilot version 4.2.2. (JSI medical systems). CNV detection was evaluated using SNPitty, which visualizes B-allele frequencies from NGS sequencing data [35]. The American College of Medical Genetics and Genomics standards and guidelines were used for interpretation of sequence variants of unknown significance (VUS) [33]. When classifying and reporting a variant we used the online software prediction program Polyphen-2 (http://genetics.bwh.harvard.edu/pph2/) and Align GVGD (http://agvgd.hci.utah.edu/agvgd_input.php) as well as the gnomAD database (https://gnomad.broadinstitute.org), cBioportal database (https://www.cbioportal.org), and the Cosmic database (https://cancer.sanger.ac.uk/cosmic).

**Table 1:** Characteristics of the custom-made targeted gene panel

Characteristics	Panel I	
Type of sample	FFPE-DNA	
Amplicon length, bp	125–175	
Amplicons designed	399 (X 2)	
Common genes included (pituitary adenoma and DTC)	1. AIP (NM_003977): exon 1–6;	
	2. BRAF (NM_004333): exon 15;	
	3. CDKN1B (NM_ 004064): exon 1–2	
	4. GNAS (NM_016592): exon 1–13	
	5. GPR101 (NM_054021): exon 1;	
	6. HRAS (NM_005343): exon 2–6;	
	7. KRAS (NM_004985): exon 2–5;	
	8. MEN1 (NM_000244): exon 1–10;	
	9. NRAS (NM_002524): exon 3;	
	10. PIK3CA (NM_006218): exon 2–21;	
	11. PRKACB (NM_207578): exon 1–10;	
	12. PRKAR1A (NM_212471): exon 2–11;	
	13. RET (NM_020975): exon 2–20;	
	14. SDHA (NM_004168): exon 2–15;	
	15. SDHAF2 (NM_017841): exon 1– 4;	
	16. SDHB (NM_003000): exon 1–8;	
	17. SDHC (NM_003001): exon 1–6;	
	18. SDHD (NM_003002): exon 1–4	
SNPs target region chromosome 11	– rs2513613	– rs10838307
	– rs34593780	– rs4267090
	– rs2631403	– rs7939803
	– rs330253	– rs2887046
	– rs73455029	– rs11233227
	– rs7949600	– rs6483324
	– rs1247726	– rs2851171
	– rs4943948	– rs736287
	– rs681017	– rs2510718
	– rs10750552	– rs1638585
	– rs1620333	– rs7110021
	– rs630172	– rs35787427
	– rs481303	– rs611697
	– rs1455113	
SNPs target region chromosome 22	– rs1970640	– rs2017869
	– rs3747031	– rs1894252
	– rs5996639	– rs956548
	– rs2285206	– rs2038010
	– rs2294206	– rs2143695
	– rs62636244	– rs5769583
	– rs17003592	– rs1296750
	– rs3884944	– rs6010046

NM and ENST are both available at http://www.ensembl.org.

*DTC* differentiated thyroid carcinoma, *FFPE* formalin-fixed, paraffin-embedded, *SNPs* single-nucleotide polymorphisms

In patient no. 17, we also examined the most common FTC driver gene alterations [36] by a targeted NGS designed to study *PTEN* and the *TERT* promoter. The panel included the entire coding sequences of *CDKN2A*, *KEAP1*, *PTEN*, *STK11*, and *TP53*, as well as hotspots: *AKT1* (exon 3), *AKT2* (3), *AKT3* (2), *ALK* (20, 22–25), *APC* (16), *ARAF* (7), *BRAF* (11, 12, 14, 15), *CDK4* (2, 4, 7, 8), *CTNNB1* (3, 7, 8), *DDR2* (14–19), *EGFR* (12, 18–21), *EIF1AX* (1, 2), *ERBB2* (*HER2*) (8, 17–21), *ERBB3* (3, 6–10, 21, 23), *ESR1* (4, 5, 7, 8), *EZH2* (16), *FBWX7* (9, 10), *FGFR1* (4, 7, 12–14), *FGFR2* (7, 9, 12), *FGFR3* (7, 9, 14, 15), *FOXL2* (1), *GNA11* (4, 5), *GNAQ* (4, 5), *GNAS* (8, 9), *HRAS* (2–4), *IDH1* (4), *IDH2* (4), *JAK2* (14), *JAK3* (4, 16), *KIT* (8, 9, 11, 13–18), *KNSTRN* (1), *KRAS* (2–4), *MAP2K1* (1–6), *MET* (2, 14, 19, 20), *MTOR* (30, 39, 40, 43, 47, 53, 56, 57), *MYD88* (5), *NFE2L2* (2), *NOTCH1* (26, 27), *NRAS* (2–4), *OXA1L* (1), *PDGFRA* (12, 14, 18), *PIK3CA* (2, 5, 8, 10, 14, 21), *POLD1* (6, 8, 12, 15–17, 24), *POLE* (9–14, 21, 25), *RAC1* (2), *RAF1* (7), *RET* (11, 16), *RHOA* (2), *RIT1* (4, 5), *RNF43* (2–10), *ROS1* (36–41), *SF3B1* (14, 15), and *SMAD4* (3, 9, 12). In addition, it also covers the known C228T, 242_243delinsTT, and the C250T of the *TERT* promoter. To investigate the presence of driver fusions, the FTC tumor of patient no. 17 was investigated using Archer technology. RNA was isolated according to manufactures instructions using the RNeasy kit (Qiagen). Subsequently, Archer was performed with the Archer FusionPlex CTL panel (Illumina) according to manufacturer’s instructions and analysed using the S5-XL system. Sequencing data were uploaded and analyzed using the Archer Analysis software (https://analysis.archerdx.com). If all quality criteria were met as indicated by the Archer’s instructions, data were considered valid. Details are available on request.

### Statistical analysis

We calculated proportions and rates for categorical variables, means ± standard deviations, or medians and ranges for parametric or nonparametric variables. For statistical analysis, the Statistical Package for the Social Sciences (SPSS) version 23.0.0 (IBM Corp, Armonk, NY, USA) was used. The significance level was set at *p* < 0.05 for all tests.

## Results

### Cohort characteristics

In total, seventeen patients were included for pathology NGS analysis. Clinical characteristics are summarized in Table 2. In most patients, the onset of thyroid carcinoma was detected later than the onset of the pituitary adenoma (median 51.5 years (IQR 48.3–66.3) versus 57.0 years (44.0–69.0)). Thyroid carcinoma was diagnosed before the pituitary adenoma in five cases, from 1 to 18 years before their pituitary adenoma had been diagnosed. Classical-variant PTC was reported in most patients (*n* = 9), following by FTC (*n* = 5) and FVPTC (*n* = 3). Thyroid carcinoma metastasis was found in five patients (29.4%); three had locoregional lymph node metastases, one had skeletal metastases, and the other had lung metastases.

**Table 2:** Clinical characteristics of patients included in the study

Characteristics	Value	
Patients from PALGA search	*n* = 14	
Type of sample available	Pituitary tumor DNA, *n* = 13 (92.9%)	
	Thyroid tumor DNA, *n* = 11 (78.6%)	
Patients with known *AIP* germline variants	*n* = 3	
Type of sample available	Pituitary tumor DNA, *n* = 2 (66.7%)	
	Thyroid tumor DNA, *n* = 3 (100.0%)	
Sex	Female/male: *n* = 15 (88.2%)/2 (11.8%)	
Age at onset pituitary adenoma (yrs)	Median, 51.5 (IQR 48.3–66.3)	
Age at onset thyroid carcinoma (yrs)	Median, 57.0 (IQR 44.0–69.0)	
No. and type of pituitary adenoma from available samples	Single, *n* = 12 (80.0%)	Multiple, *n* = 3 (20.0%)
	Nonfunctioning, *n* = 5 (33.3%)	FSH + LH, *n* = 1 (6.7%)
	ACTH, *n* = 2 (15.0%)	FSH + TSH, *n* = 1 (6.7%)
	GH, *n* = 2 (15.0%)	GH + PRL, *n* = 1 (6.7%)
	LH, *n* = 1 (6.7%)	
	PRL, *n* = 1 (6.7%)	
	Unknown, *n* = 1 (6.7%)	
No. and type of thyroid carcinoma from available samples	Single, *n* = 14 (100.0%)	
	PTC, *n* = 7 (50.0%)	
	FTC, *n* = 4 (28.6%)	
	FVPTC, *n* = 3 (21.4%)	
Metastasis	*n* = 5 (29.4%)	

*ACTH* adrenocorticotropic hormone, *FSH* follicle-stimulating hormone, *FTC* follicular thyroid cancer, *FVPTC* follicular variant of papillary thyroid carcinoma, *GH* growth hormone, *IQR* interquartile range, *LH* luteinizing hormone, *SS* Sanger sequencing, *TSH* thyroid-stimulating hormone, *PA* pituitary adenoma, *PRL* prolactin, *PTC* papillary thyroid carcinoma, *TC* thyroid carcinoma, *yrs* years

Regarding the pituitary adenomas, no pituitary hormonal staining was reported in most patients (i.e., NFPAs; *n* = 5), while others stained positively for ACTH (*n* = 2), GH (*n* = 2), LH (*n* = 1), and PRL (*n* = 1). Combined expression was reported in three patients: GH and PRL, and FSH with either LH, or TSH. The staining data were not reported in two patients.

### Genetic characterization

#### Detection of variants in sporadic patients

**Fig. 1:**
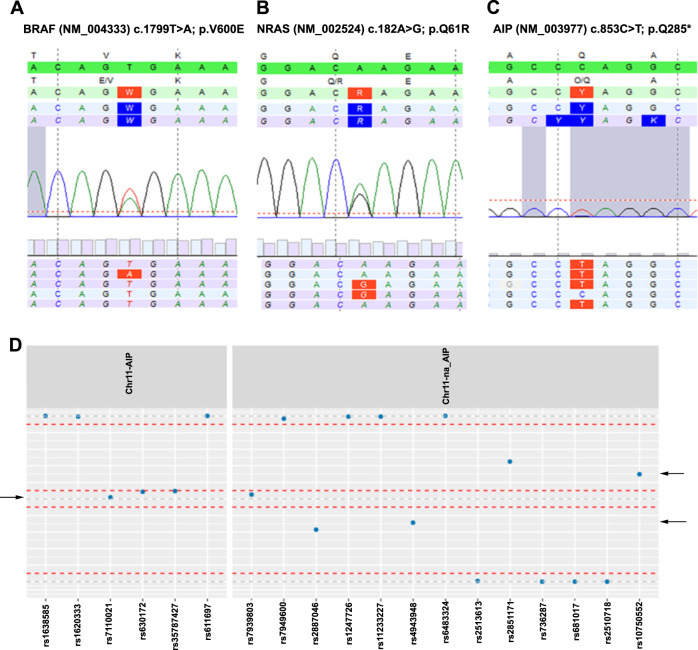
Direct sequencing of PCR antisense products in thyroid tumor samples obtained from 14 patients revealing the presence of (**a** corresponding with patient no. 2) the *BRAF* p.V600E variant in six patients, (**b** corresponding with patient no. 1) the *NRAS* p.Q61R variant in two patients, and (**c** corresponding with patient no. 17) the *AIP* p.Q285 variant in one patient. LOH of chromosome 11 was identified in two of the 15 pituitary tumor samples; one was a partial chromosome 11 LOH deletion. In thyroid tumor samples, in 2 of the 14 samples chromosome 11 was identified; one was a partial chromosome 11 LOH deletion. Chromosome 22 was identified in two of the 14 thyroid tumor samples. (**d** arrow: corresponding with patient no. 12) Demonstrates a representative example of LOH. LOH loss of heterozygosity

NGS analysis of the 14 patients from the PALGA search revealed no known mutations in targeted genes in pituitary tumor DNA and eight mutations in thyroid tumor DNA. Table 3 summarizes the identified mutations and CNVs (i.e., LOH) of chromosome 11 and 22. The 13 pituitary tumor samples showed no gene mutations. Among the 11 thyroid tumor samples, *BRAF* (5/11, 45.5%) was the gene most frequently mutated, followed by *NRAS* (3/11, 27.3%). These classical *BRAF* (p.V600E) point mutation were found in 57.1% (*n* = 4) of classical-variant PTC specimen and once (33.3%) in FVPTC specimen (Fig. 1a). *NRAS* codon 61 point mutation is the most common among *RAS* mutations and this was only observed in FTC specimen: p.Q61R twice (50.0%) (Fig. 1b) and p.Q61K once (25.0%).

**Table 3:**
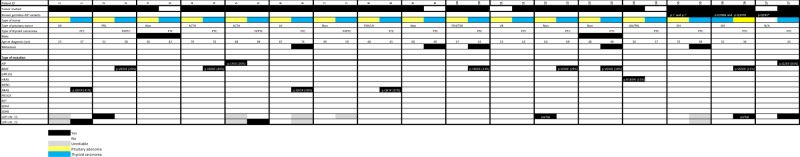
Cluster of mutations and CNVs

Cases are categorized by pituitary adenoma and differentiated thyroid carcinoma

*ACTH* adrenocorticotropic hormone, *FSH* follicle-stimulating hormone, *FTC* follicular thyroid cancer, *FVPTC* follicular variant of papillary thyroid carcinoma, *GH* growth hormone, *LH* luteinizing hormone, *LOH* loss of heterozygosity, *Non* nonfunctioning pituitary adenomas, *TSH* thyroid-stimulating hormone, *PA* pituitary adenoma, *PRL* prolactin, *PTC* classical-variant papillary thyroid carcinoma, *TC* thyroid carcinoma, *UK* unknown, *VUS* variant of unknown significance, *yrs* years

In addition, two VUSs were found in pituitary tumor DNA. These VUSs involved *AIP*-variant c.433C>T; p.145S (*n* = 1) and *HRAS*-variant c.505C>T; p.R169W (*n* = 1) (Table 3). Prediction software to determine pathogenicity predicted the *AIP* p.145S variant as benign (Align GVGD Class C0) to probably damaging (Polyphen-2 score of 0.978 (sensitivity: 0.76; specificity: 0.96)). The variant was never detected in the healthy population (gnomAD), nor is it found in large series of different tumor types from the cBioportal (*n* = 10,967 tumor samples) and Cosmic (*n* = 92,857 tumor samples) databases. Therefore, we considered *AIP* p.145S as a VUS. The prediction software predicted *HRAS* p.R169W as probably damaging (GVGD Class C15 and a Polyphen-2 score of 0.988 (sensitivity: 0.73; specificity: 0.96)). However, the variant also appeared in the European and American population with an allele frequency of 0.01% (rs151229168; gnomAD). In addition, a TCGA PanCancer Atlas Studies search using the cBioportal database did not report the *HRAS* p.R169W variant in the 10,967 tumor samples. Furthermore, the variant is also not reported by the Cosmic database in all tumor types, including thyroid tumors (cBioportal 500 and Cosmic 9985 thyroid samples). So, although the prediction software indicates the *HRAS* variant as probably damaging, we consider *HRAS* p.R169W as a VUS.

LOH of chromosome 11 was identified in two of 13 pituitary tumor samples (15.4%), both in 11q13; one had a partial chromosome 11 LOH deletion (Table 3). A representative example of LOH is demonstrated in Fig. 1d. No pituitary tumor samples showed LOH of chromosome 22. Out of the 11 patients with thyroid carcinomas, two patients had LOH of chromosome 22. No LOH of chromosome 11 was identified in the thyroid carcinomas.

#### Detection of variants in patients with known germline AIP variants

Genetic screening of germline DNA from patients 15, 16, and 17 revealed several *AIP* variants. Patient no. 15 had two *AIP*-variants: c.787 + 25 G>A; p.? and *60 G>C; p.?. Variant prediction software noted *60 G>C as probably benign, whereas c.787 + 25 G>A was noted in 2/4 prediction models to lead to a new splice acceptor site at c.787 + 27. In the second patient (patient no. 16), two *AIP*-variants were detected: c.682 C>A; p.Q288K, which is a known benign polymorphism, and c.920 A>G; p.Q307R; considered a benign variant. In patient no. 17, a pathological *AIP*-variant c.853 C>T; p.Q285* was identified.

NGS analysis of the three patients with known germline *AIP* variants revealed no known mutations in the pituitary tumor DNA, however, two mutations were identified in the thyroid tumor DNA. In patient no. 17, the *AIP*-variant c.853 C>T; p.Q285* was confirmed in FTC specimen (allele frequency 83%), while no mutations in other genes or translocations were observed (Fig. 1c). The *BRAF* (p.V600E) point mutation was found in patient no. 16. No pituitary tumor samples showed LOH of chromosome 11. LOH of chromosome 11 was identified in two (patient no. 16 and 17) of the three thyroid carcinomas (66.7%); one was a partial chromosome 11 deletion. No LOH of chromosome 22 was identified in both pituitary adenomas and thyroid carcinomas.

## Discussion

To our knowledge, this is the first study to analyze the prevalence of *AIP* gene mutations and mutations in genes that have been associated with neuroendocrine tumors in series of tumors from patients presenting with both pituitary adenomas and DTCs. We showed that genetic alterations were observed in 71.4% (10/14) of DTCs and in 13.3% (2/15) of pituitary adenomas tissues, while there was no overlap between genetic alterations within tissues from the same patient. Among patients with pathological germline *AIP* variants, one *AIP* variant c.853 C>T; p.Q285* was confirmed in the FTC specimen (patient no. 17), including evidence of loss of the *AIP* wild-type allele, based on the relatively high allele frequency (83%) of the germline mutation in the tumor DNA. Unfortunately, we were unable to confirm this LOH based on the SNPs analysis, due to low quality of the FTC tissue. This patient came from an *AIP*-mutated FIPA kindred, however, her pituitary gland was unaffected. This supports that the finding of DTCs and pituitary adenomas are not totally fortuitous coexistence in an *AIP* mutation-positive FIPA kindred, thereby echoing a recent finding of FTC in an *AIP* mutation carrier by Daly et al. [28]. In a second patient with a somatotropinoma with two benign *AIP*-variants (p.Q288K and p.Q307R), a somatic *BRAF* (p.V600E) mutation was detected in PTC specimen in combination with a partial chromosome 11 LOH deletion. Although the partial chromosome 11 LOH deletion could indicate a second hit in the thyroid tissue, the observed LOH concerns SNPs located downstream (3′) of the *AIP* gene, while the SNPs located in the *AIP* gene did not indicate LOH.

It is noteworthy that although the most common mechanism to lose the wild-type copy of a tumor suppressor gene (e.g., *AIP*) in DTC specimen is a large deletion affecting the wild-type allele, other mechanisms could also play a role, such as another somatic mutation in other parts of the gene, or silencing of the wild-type copy with epigenetic mechanism-promoter methylation or microRNAs which are not covered by NGS. Moreover, we should emphasize that DTCs are more progressed in transformation since they are malignant when compared with pituitary adenomas. Therefore, it might be interesting to investigate the role of *AIP* mutation in thyroid adenomas (i.e., earlier in the transformation) in further studies.

In the total cohort, the most common oncotype in pituitary adenoma-related DTC was classical-variant PTC (9 out of 14 cases; see Table 2) with a high frequency (42.9%, 6/14) of *BRAF* (p.V600E) mutations, whereas none of these cases harbored *NRAS* mutations. These results confirm and build upon previous studies stating that among PTC, virtually all tumors that harbor a *RAS* mutation grow forming neoplastic follicles and no papillary structures and are, therefore, diagnosed as the FVPTC, while *BRAF* is the most frequent genetic alteration in classical-variant PTC [15, 16]. In line with this, the *NRAS* codon 61 point mutations were only observed in FTC specimen in three cases (21.4%) [37], which was the second most frequently mutated gene among the thyroid tumor samples. Although the limited number of DTCs in the present series prevents us from drawing any final conclusions on the prevalence of *BRAF* and *NRAS* mutations in DTCs in patients with versus those without pituitary adenomas, *BRAF* and *NRAS* seems the main genetic drivers of thyroid follicular epithelial cell transformation in our cases.

Our results are not in accordance with previous data, which suggested that *BRAF* mutation may not play a dominant role in development of DTC in patients with acromegaly [11, 38]. In these studies only one (9.1%) [11] or two (14.3%) [38] patients with concomitant PTC had the *BRAF* mutation, which in both studies was more frequently present in PTC patients without acromegaly. This discrepancy might be explained by [1] the inclusion of relative more FVPTC patients in the study from Aydin et al. [38] which is different to our cohort, or [2] our distinct study population, as included patients had not only of GH-producing adenomas but all five hormone-secreting cell types. In fact, previous studies [11, 29, 38] were carried out exclusively in acromegaly patients, while the patients we studied included only one patient with a GH-producing tumor. Therefore, direct comparison between our cohort and the acromegaly cohorts is limited.

In line with our findings, studying 12 DTC patients with acromegaly, Mian et al. reported that 70% of PTC patients with acromegaly were *BRAF* positive [29]. Moreover, *AIP* expression was similar between neoplastic and normal tissue, while the aryl-hydrocarbon receptor (AHR) was expressed more in PTCs carrying *BRAF* mutations than in normal tissue, irrespective of acromegaly status [29]. These data suggest that *BRAF* mutations and AHR overexpression may be associated with DTC risk in acromegaly, at least in patients with concomitant PTC.

Although there is no gender preponderance in pituitary adenoma patients, the vast majority of those with concomitant DTCs were female (15 out of 17) and is in accordance with previous literature, probably reflecting a trend seen in the general population. When comparing differences between patients with and without concomitant DTC, ist seems the former were relatively older. The mean age at onset and diagnosis of pituitary adenoma was mean 55 years [SD 12] in the cohort vs. mean 44 years [SD 17] in the general population, with the mean age at diagnosis in female patients being younger; 34 years [39]. The onset and diagnosis of DTC was median 57 years [IQR 44–69] in the cohort vs. 46 years [IQR 10–85] in the general population [40], with the median age at diagnosis in female patients being younger; 45 years [40]. In addition, in the two previously reported cases of acromegaly and concomitant PTC, and harboring a germline *AIP* variant, both patients were female and diagnosed with acromegaly at age 67 and 74, respectively.

This is in contrast to the clinical characteristics of patients bearing germline *AIP* mutations; the disease usually manifests in the second decade of life, almost all cases are diagnosed before the age of 30 years [28, 41–44] and they are predominantly males [45]. With this in mind, it should be stressed that after progress is made in the treatment of pituitary adenomas and its complications, these patients may live long enough to reach the age of increased cancer risk.

Strength of our study lies in the relatively large number of patients in which the pituitary adenoma and concomitant DTC tumor tissue were systematically investigated by targeted NGS. The main limitations of our study lie in the retrospective collection of tumor samples, and we had to exclude several tissues due to low quality. Another limitation is the lack of clinical data from the patients, including follow-up and family history data. Therefore, it should be stressed that we cannot rule out if patients from the PALGA search had additional risk factors for DTCs (e.g., received radiotherapy). At last, we should be borne in mind that the increased number of the diagnoseis of thyroid cancer in these patients could be due to the fact that they are examined more accurately and more frequently than before (i.e., surveillance bias).

In conclusion, the absence of somatic *AIP* mutations observed in patients with pituitary adenomas and concomitant DTCs suggest that their contribution to tumoral pathogenesis is probably limited and seems unlikely the genetic cause predisposing to the higher DTC risk observed in these patients. Though the finding of DTCs and pituitary adenomas could represent a new variant of MEN syndrome with a de novo germline mutation in a not yet identified gene, we suggest that this may be a fortuitous coexistence based on our observed variants that were similar to those of sporadic DTCs. In view of this and in line with the clinical practice guidelines from Katznelson et al. [46], we recommend including regularly thyroid examination and thyroid ultrasound only if there is a palpable thyroid nodularity. While the finding of the *AIP*-variant and LOH at this locus in FTC specimen in one *AIP* mutation-positive case, opens up a potential role for *AIP* mutation as an initiating event, further studies of *AIP* genetic status among DTCs in FIPA kindred cohorts are warranted to answer this question.

## Supplementary information

Supplementary appendix
