# Supplementary material for: The role of AIP variants in pituitary adenomas and concomitant thyroid carcinomas in the Netherlands: a nationwide pathology registry (PALGA) study
Source: Endocrine. 2020 Apr 24;68(3):640–9. doi: 10.1007/s12020-020-02303-7 (PMC7308253; doi:10.1007/s12020-020-02303-7)
Supplement: Supplementary file 1 — Supplementary appendix [file 12020_2020_2303_MOESM1_ESM.docx]

**Supplementary appendix**

The link to the website for each database.

1. dbSNP
Link to website: <https://www.ncbi.nlm.nih.gov/snp/>

2. 1000Genomes
Link to website: <https://www.internationalgenome.org/>

3. ESP

Link to website: <https://evs.gs.washington.edu/>

4. GoNL

Link to website: <http://www.nlgenome.nl/>

5. ClinVar

Link to website: <https://www.ncbi.nlm.nih.gov/clinvar/>

6. LOVD

Link to website: <https://www.lovd.nl/>

7. UCSC

Link to website: <https://genome.ucsc.edu/>

8. cBioportal

Link to website: <http://www.cbioportal.org/>

9. JAX
Link to website: <https://ckb.jax.org/>

10. Cosmic
Link to website: <https://cancer.sanger.ac.uk/cosmic>
